# Treadmill Training Effect on the Myokines Content in Skeletal Muscles of Mice With a Metabolic Disorder Model

**DOI:** 10.3389/fphys.2021.709039

**Published:** 2021-11-10

**Authors:** Anna Nikolaevna Zakharova, Tatiana Alexandrovna Kironenko, Kseniia G. Milovanova, A. A. Orlova, E. Yu Dyakova, G. Kalinnikova Yu, Anastasia V. Kabachkova, Alexander V. Chibalin, Leonid V. Kapilevich

**Affiliations:** ^1^Department of Sport Tourism, Sport Physiology and Medicine, National Research Tomsk State University, Tomsk, Russia; ^2^Department of Molecular Medicine and Surgery, Section of Integrative Physiology, Karolinska Institutet, Stockholm, Sweden; ^3^Central Research Laboratory, Siberian State Medical University, Tomsk, Russia

**Keywords:** myokines, cytokines, muscles, running load, diabetes mellitus, biorhythms

## Abstract

The effect of treadmill training loads on the content of cytokines in mice skeletal muscles with metabolic disorders induced by a 16 week high fat diet (HFD) was studied. The study included accounting the age and biorhythmological aspects. In the experiment, mice were used at the age of 4 and 32 weeks, by the end of the experiment—respectively 20 and 48 weeks. HFD feeding lasted 16 weeks. Treadmill training were carried out for last 4 weeks six times a week, the duration 60 min and the speed from 15 to 18 m/min. Three modes of loading were applied. The first subgroup was subjected to stress in the morning hours (light phase); the second subgroup was subjected to stress in the evening hours (dark phase); the third subgroup was subjected to loads in the shift mode (the first- and third-weeks treadmill training was used in the morning hours, the second and fourth treadmill training was used in the evening hours). In 20-week-old animals, the exercise effect does not depend on the training regime, however, in 48-week-old animals, the decrease in body weight in mice with the shift training regime was more profound. HFD affected muscle myokine levels. The content of all myokines, except for LIF, decreased, while the concentration of CLCX1 decreased only in young animals in response to HFD. The treadmill training caused multidirectional changes in the concentration of myokines in muscle tissue. The IL-6 content changed most profoundly. These changes were observed in all groups of animals. The changes depended to the greatest extent on the training time scheme. The effect of physical activity on the content of IL-15 in the skeletal muscle tissue was observed mostly in 48-week-old mice. In 20-week-old animals, physical activity led to an increase in the concentration of LIF in muscle tissue when applied under the training during the dark phase or shift training scheme. In the HFD group, this effect was significantly more pronounced. The content of CXCL1 did not change with the use of treadmill training in almost all groups of animals. Physical activity, introduced considering circadian rhythms, is a promising way of influencing metabolic processes both at the cellular and systemic levels, which is important for the search for new ways of correcting metabolic disorders.

## Introduction

Type 2 diabetes mellitus (DM II) contributes to the alarming increase of chronic diseases worldwide, accounting for about 90% of diabetes cases (Heimro et al., 2021). Its pathogenesis is associated with insulin resistance in peripheral tissues and with an increase in blood glucose concentration (Groop and Eriksson, 1992; Højlund, 2014; Fujimaki and Kuwabara, 2017).

Physical activity reduces cardiometabolic risk, and physical inactivity increases the risk of chronic metabolic diseases (Zhao et al., 2021). Exercise-induced muscle contraction improves metabolic regulation in all tissues. Skeletal muscles, through the release of endocrine factors (myokines), interact with metabolic organs such as adipose tissue, liver and pancreas (Pedersen and Febbraio, 2008; Brandt and Pedersen, 2010; Pedersen and Saltin, 2015). Recent studies provide evidence to suggest that a number of myokines are capable of modulating adipose tissue metabolism and thermogenic activity, endogenous liver glucose production and β-cell insulin secretion (Laurens et al., 2020). This new paradigm offers a compelling hypothesis and molecular basis to explain the link between physical inactivity and chronic disease.

The effects of exercise depend on the concerted action of a variety of responses, many of which are controlled by circadian rhythms (Cedernaes et al., 2018; Mancilla et al., 2020; Savikj and Zierath, 2020; Harris and Kuo, 2021). However, little is known about the molecular effect of the time of day on a single exercise performance. Both mice and humans have been shown to exhibit differences in exercise ability at different times of the day (Ezagouri et al., 2019; Savikj et al., 2019). Glycolytic activation is characteristic of exercise in the early active phase in mice, that is, the time of day is a critical factor for enhancing the positive effects of exercise on both metabolic pathways in skeletal muscle and systemic energy homeostasis (Sato et al., 2019).

Muscle tissue is the main consumer of energy, of fatty acids and glucose (Meneilly, 2001; Frontera and Ochala, 2015). However, opposite to the effect of physical activity, the content in the blood plasma of a number of cytokines, including the tumor necrosis factor TNF-α, interleukins IL-1β, IL-6, IL-8, IL-15, and leukemia inhibitory factor (LIF) (Sprenger et al., 1992; Drenth et al., 1995; Nehlsen-Cannarella et al., 1997; Ostrowski et al., 1999; Petersen et al., 2005). Skeletal muscles capable of producing IL-6, its content in blood plasma was increased after physical exertion (Steensberg et al., 2000). This fact was confirmed by the detection of IL-6 mRNA transcription in the nuclei of muscle cells isolated from biopsies of human muscles after a single exercise (Keller et al., 2001). In the next decade, the idea of the endocrine function of skeletal muscles was formed, due to which they are able to produce cytokines and other peptides (Pedersen and Febbraio, 2012; Kapilevich et al., 2015, 2017). Many researchers designate these compounds as myokines, noting their ability to exert various physiological effects (Pedersen and Febbraio, 2008; Iizuka et al., 2014; Kapilevich et al., 2016).

Myokines, through anti-inflammatory effects on the muscles themselves, can counteract insulin resistance (Brandt and Pedersen, 2010). In addition, during exercise, protein molecules are expressed that are involved in glycolysis and amino acid metabolism (Hansen et al., 2015). Physical activity has a natural anti-inflammatory effect and improves metabolism (Karstoft and Pedersen, 2016). Therefore, skeletal muscle tissue can play a significant role in the correction of metabolic disorders. Due to the increasing number of recently identified molecules secreted by muscles (nucleic acids, peptides), the concept of muscle secretion is constantly evolving. Starting from the initial concept of myokines (they were also called muscle cytokines) to the coverage of a wider pool of molecules (Aguer et al., 2020). Many authors admit that the differentiation of sources of cytokine production in muscle tissue is difficult, these sources can be both muscle cells and other types of cells, including macrophages infiltrating muscle tissue or other immune cells (Pillon et al., 2013).

Myokines including IL-6, IL-15, whose blood levels change after exercise, are able to modulate muscle metabolism, and can also affect other tissues to adapt to energy needs. The concentration of myokines in the blood after exercise in people with diabetes differs from that in healthy volunteers. In research Garneau et al. (2020) it was found that plasma IL-8 and SPARC levels were reduced in the group of women with obesity, whereas plasma IL-13 concentrations were elevated in comparison to non-obese women both before and after the exercise bout. Also was found that plasma FGF21 concentration during the 24 h following the bout of exercise was regulated differently in the non-obese in comparison to obese women. Plasma concentrations of FGF21, IL-6, IL-8, IL-15, and IL-18 were regulated by acute exercise. This results confirm the results of others concerning exercise regulation of circulating myokines while providing insight into the time course of myokine release in circulation after an acute exercise bout and the differences in circulating myokines after exercise in women with or without obesity (Garneau et al., 2020).

One of the models for the formation of DM II is keeping animals on a high-fat diet. A HFD feeding can lead to obesity, hyperinsulinemia, and altered glucose homeostasis due to inadequate islet compensation (Winzell and Ahren, 2004) and skeletal muscle insulin resistance (Galuska et al., 2009). Since obesity in this case is caused by food manipulation rather than cytotoxic substances, it is believed that such models are more similar to the disease in humans. Obesity induced by a high-fat diet has been shown to reduce skeletal muscle myokines in rats (Ahn and Kim, 2020). At the same time, combined aerobic exercise and resistance exercise increased the secretion of myokines in the skeletal muscles of obese rats and helped to reduce inflammation (Ahn and Kim, 2020).

The aim of our study was to investigate the effect of treadmill training on the content of cytokines in skeletal muscles of mice with a model of metabolic disorders induced by a HFD, considering age and biorhythmological aspects.

## Research Methodology

C57bl/6 male mice were used in the study. The mice were obtained from the vivarium of the Tomsk National Research Medical Center of the Russian Academy of Sciences. Mode of keeping animals: day/night: 12/12 h, daylight hours start at 6:00, free access to food and water, room temperature 24°C. The study was carried out in accordance with the principles of the Basel Declaration and was approved by the Bioethics Commission of the Biological Institute of Tomsk State University (Protocol No. 32 dated 02.12.2019).

**FIGURE 1:**
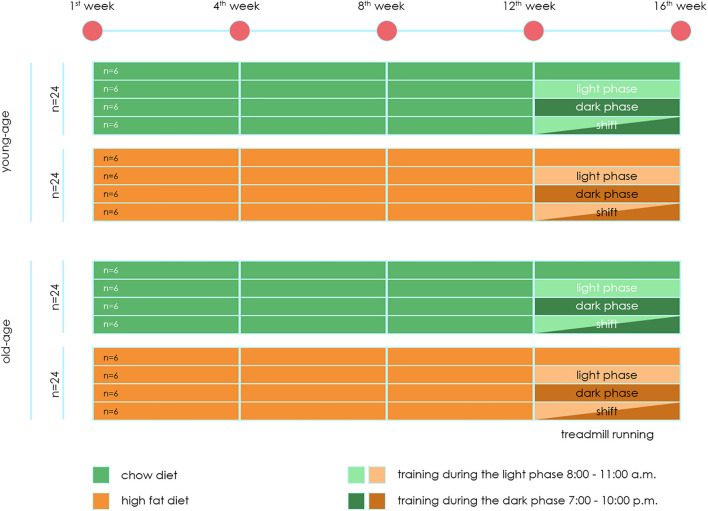
Experiment design.

A total of 96 mice were included in the experiment. 48 mice at the beginning of the experiment have age 4 weeks old (young-age mice group). 48 mice at the beginning of the experiment have age 32 weeks old (old-age mice group). Experiment design is present on Figure 1.

Each experiment lasted 16 weeks. Up to 12 weeks, mice were divided into two groups:

- Animals fed the Chow diet (CHD) – 24 mice in each age per group.
- Animals fed the High fat diet (HFD) – 24 mice in each age per group.

The first group ate food for laboratory animals ‘‘Prokorm’’ (JSC ‘‘Biopro,’’ Novosibirsk^1^): wheat, barley, bran, corn gluten, fish flour, protein feed mixture, sunflower oil, soybean meal. Caloric content 3,000 kcal/kg, including 18% of calories from fat.

To form a model of metabolic disorders, a HFD was used, which was developed by the team specifically for this experiment. The composition and energy value of the feed were described previously (Kapilevich et al., 2019). The composition of the feed is given in Table 1. The products were ground in a blender into a homogeneous mixture, after which the mass was formed into granules up to 10 mm in diameter and dried in an oven at 300°C. The food was cooked for 5 days and stored at −20°C. Mice were fed the same diet for a total of 16 weeks.

**TABLE 1:** Characteristics of diets for the experimental (High fat diet) and control groups (Chow diet).

**Characteristics**	**High fat diet**	**Chow diet**
Caloric content, kcal/kg	5,100	3,000
Including% of calories from fat	59%	2,5%
Composition		
Fats	33,3%	6%
Including animal fats	25%	-
Carbohydrates	17%	3,6%
Protein	13%	23,9%
Lysine	0,8%	1,5%
Methionine + Cysteine	0,5%	0,9%
Macronutrients		
Calcium	0,9%	1%
Phosphorus	0,7%	0,8%
Sodium chloride	0,24%	0,34%
Vitamins and minerals	+	+
Antioxidant, amino acids	+	+

The use of this HFD in mice leads to an increase in body weight and the development of obesity, hyperglycemia, decreased glucose tolerance and hyperinsulinemia (Kapilevich et al., 2019). Starting from the 12th week, each animal group was divided into two subgroups – exposed to (main – 18 mice) and not subjected (control – six mice) treadmill training loads of animals.

**FIGURE 2:**
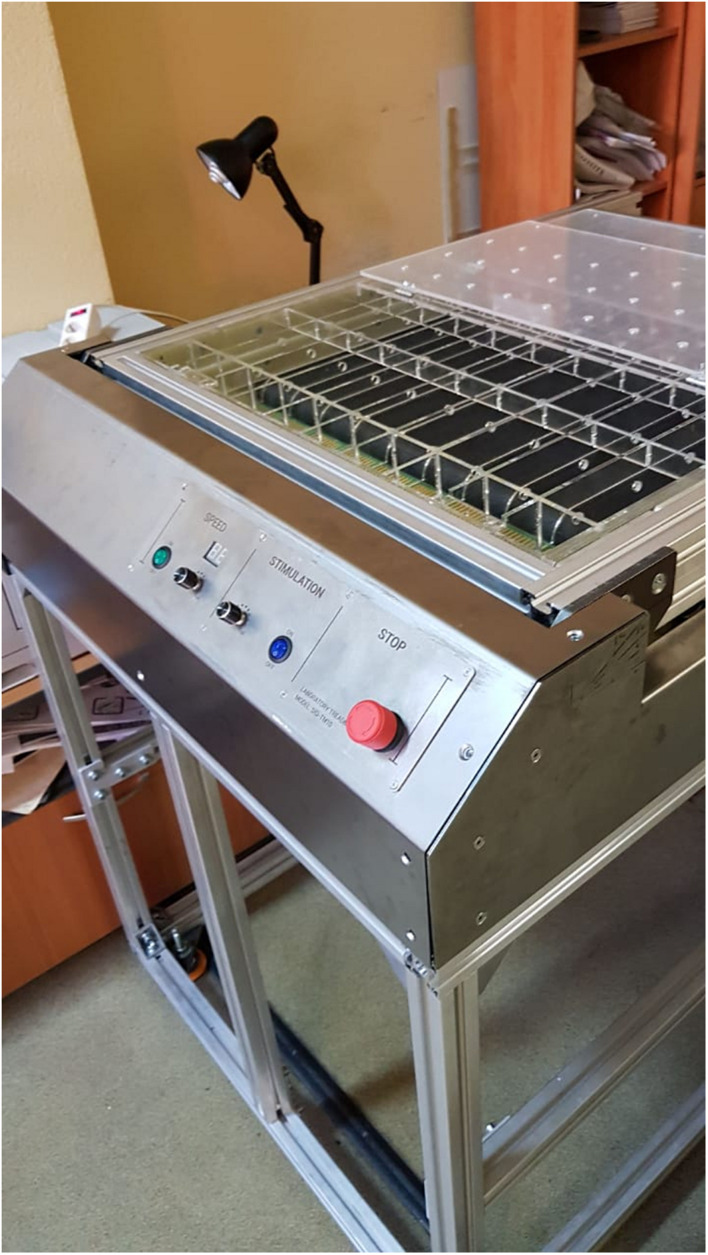
BMELAB SID-TM10 treadmill.

To normalize the load, we used a BMELAB SID-TM10 treadmill (Figure 2; Zakharova et al., 2020). There were 10 animals on the treadmill at the same time; they were separated from each other by transparent walls. The compulsion to run is carried out by electrical stimulation.

**FIGURE 3:**
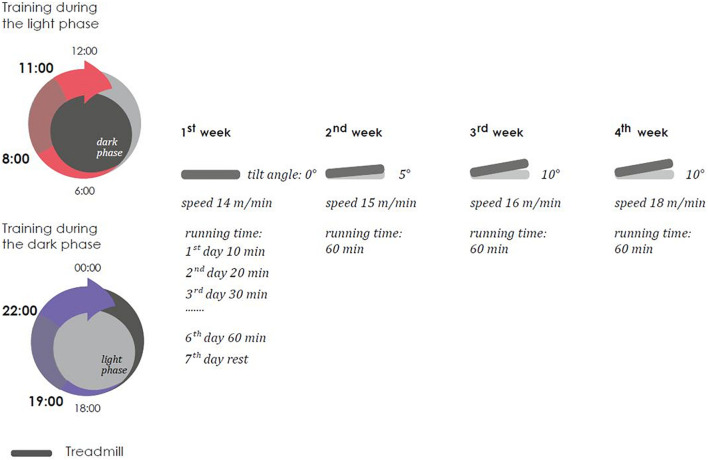
Scheme of treadmill training.

Treadmill training were carried out for 4 weeks six times a week, the duration of the load gradually increased during the first 6 days from 10 to 60 min (an increase of 10 min per day) and did not change more over the next 3 weeks. Every week, the angle of the treadmill’s incline (from 0 to 10°) and the speed of its rotation (from 15 to 18 m/min) were changed. The training sessions were not performed once a week (on the 7th day). Scheme of treadmill training is present on Figure 3.

Three modes of loading were applied. The first subgroup was subjected to stress in the morning hours from 8-00 to 11-00 (light phase). The second subgroup was subjected to stress in the evening hours [from 19-00 to 22-00 (dark phase), the training was performed in a dark room, illumination with diffuse red light of low intensity was allowed]. The third subgroup was subjected to loads in the shift mode. The first- and third-weeks treadmill training was used in the morning hours, the second and fourth treadmill training was used in the evening hours. It is important to note that the dark phase is the active phase for mice, and the light phase is the sleep phase.

The sacrificing of experimental animals was carried out by decapitation method 24 h after the last load. Mice young-age group have 20-week-old, mice old-age group have 48-week-old. M. gastrocnemius was dissected from both hind limbs, muscle tissue was cleaned of connective and adipose tissue and frozen in liquid nitrogen. The collected samples were stored in a freezer at a temperature of −80°C.

A tissue sample weighing 18 mg was separated in the cold, the tissue was crushed into smaller fragments and placed in a test tube with lysis buffer and three iron beads for homogenization, 0.3 mm in size. Lysis buffer composition: NaCl - 137 mM, KCl - 2.7 mM, MgCl2 - 1 mM, Triton X-100 - 1%, Glycerol - 10%, Tris pH 7.8 - 20 mM, EDTA – 1 mM, DTT - 1 mM, Na pyrophosphate - 5 mM, Benzamidine - 1 mM, Leupeptin - 1 mkg/ml, PMSF - 0.2 mM, NaF - 10 mM.

Samples were homogenized at 2,850 rpm for 15 min using a TissueLyser LT laboratory homogenizer (Qiagen, Germany). Then, the tubes with samples, lysis solution and three homogenization beads were placed on a shaker and incubated (lysed) for 1 h at 4°C. After the specified time, the balls were removed from the tube using a magnet, all procedures were carried out in the cold, and then the tubes with samples were centrifuged at 10,000 *g* for 5 min at 4°C.

To determine the total protein in the sample, the Bradford method was used. The concentration of cytokines in the solution was determined by the method of enzyme-linked immunosorbent assay (ELISA). We used ELISA kits with antibodies to the corresponding proteins: interleukin 6 (IL-6), neutrophil activating protein 3 (Neutrophil Activating Protein 3 - NAP3, aka chemokine ligand 1 - CXCL-1), leukemia inhibiting factor (LIF) - Platinum ELISA Kit (eBioscience, Austria); interleukin 15 (IL-15) - RayBio^®^ IL-15 ELISA Kit (RayBio^®^, United States).

The glucose tolerance test (GTT) was performed before and after training. To test for glucose tolerance, mice were not given food for 4 h, while maintaining free access to water, in the morning the animals were weighed and blood glucose was determined (0 min). Then the animals were injected intraperitoneally with a solution of 40% glucose (2 g/kg) (carbohydrate load). The calculation of the glucose dose was carried out in accordance with the body weight of each animal. The blood glucose level was determined 15, 30, 60, and 120 min after the carbohydrate load (Nagy and Einwallner, 2018).

Measurement of blood glucose concentration was carried out using a portable glucometer PKG-02.4 Satellite Plus (LLC “Company” ELTA, Russia). Blood samples were obtained by puncture of the tail vein.

### Statistical Analysis

The obtained results were analyzed using GraphPad Prism 7 (GraphPad Software) and the statistical analysis package STATISTICA 8.0. The Shapiro–Wilk test was used to determine the normality of distribution. Three-way ANOVA was used to compare the studied groups. Whenever the assumptions of normality did not hold, Kruskal–Wallis test followed was performed. If main effects were statistically significant, *post hoc* Bonferroni correction was performed. Continuous variables are presented as means and standard deviations. The value of *p* < 0.05 was considered statistically significant.

## Results

The blood glucose concentration in groups of mice. In the young-age mice group at the 16th week of the experiment at rest, the glucose level in the chow diet group was statistically significantly lower (6.03 ± 0.5 mmol/l) than in the HFD group (7.21 ± 0.3 mmol/l). In the chow diet group, the highest glucose level 2 h after the carbohydrate load was found in the group of mice not exposed to physical activity (7.94 ± 0.8 mmol/l). Also, a high glucose level was noted in the light and dark phase groups (6.66 ± 0.7 and 7.11 ± 0.3 mmol/l). In the shift training group, the glucose level was the lowest (6.18 ± 0.5 mmol/l). In the HFD group, the highest glucose level was observed in the control group of mice and in the shift training group (11.24 ± 1.2 mmol/l; 11.33 ± 1.5 mmol/l). At the same time, in the light (9.88 ± 1.4 mmol/l) and dark (8.35 ± 1.1 mmol/l) phase groups, the glucose level was statistically significantly lower than in the control group.

In the group of aged mice at the 16th week of the experiment at rest, the glucose level in the chow diet group was statistically significantly lower (5.22 ± 0.9 mmol/l) than in the HFD group (7.04 ± 0.3 mmol/l). The highest glucose levels were found in the non-exercise group (7.07 mmol/l). At the same time, in groups of mice with physical exercise, the concentration is 28% lower. No differences were found between groups with different training regimes. After 120 min the carbohydrate load in the HFD group, the glucose concentration was 19.1 ± 1.3 mmol/l), which is two times higher than the glucose concentration in all groups of mice that were exposed to physical exertion. There were no differences between the groups with different training regimes.

Before the start of the experiment, the 20-week-old mice had a body weight of 19.8 ± 0.5 g, and the 48-week-old mice - 31.4 ± 0.6 g. At the 12th week of the experiment, the young-age mice who received HFD, body weight increased to 35.5 ± 1.0 g (*p* < 0.05), and in those who received CHD – to 27.7 ± 1.0 g (*p* < 0.05). In the old-age mice group at the 12th week of the experiment, body weight increased to 39.4 ± 1.0 g (*p* < 0.05) in those who received HFD and to 32.7 ± 0.8 g (*p* < 0.05) in CHD.

**FIGURE 4:**
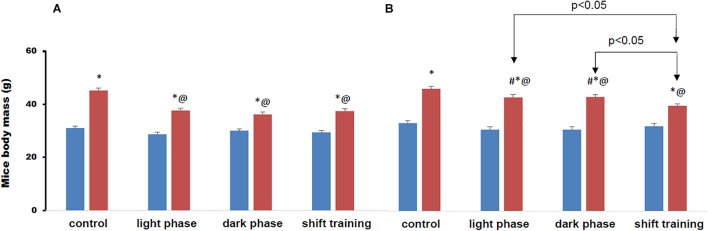
Mice body mass on the 16-th week of the experiment (grams). Panel **(A)** shows young-age mice. Panel **(B)** shows old-age mice. Blue columns represent chow diet and red columns represent high fat diet. Data are presented as the mean ± error of the mean. *Significantly different (*p* < 0.05) from Chow diet. #Significantly different (*p* < 0.05) from young-age mice. @Significantly different (*p* < 0.05) from control.

The body weight of animals at the 16th week of the experiment is shown in Figure 4. Regular physical activity in the form of treadmill training contributed to a significant decrease in body weight in animals fed a HFD. However, they remained above the CHD group. In the group of young mice, the decrease in body weight by the 16th week did not depend on the training time. There were no differences in body weight between groups of young mice in all groups with physical load. In the group of 48-week-old, the decrease in body weight in mice with the shift training regime was the greatest. The body weight in this group decreased by 13.3%, while the groups of mice light phase and dark phase the weight decreased by only 6%.

In the groups of animals fed the high fat diet, the excess body weight over the group that received the chow diet was observed from the 8th week in young animals, and already from the 4th week in the old animals (Figure 4). Regular exercise contributed to a significant decrease in body weight in animals fed a high fat diet, but they remained above the values in the group receiving a chow diet.

In the group of young mice, the decrease in body weight by the 16th week did not depend on the training time. However, in the group with the Shift training, the effect manifested itself earlier (Figure 4A). In the group of old-age mice, the decrease in body weight in mice with the Shift training was the greatest. In the chow diet group, forced jogging also led to a decrease in body weight, but to a much lesser extent than in the high fat diet group of mice (Figure 4B).

The results obtained are in good agreement with the data of a number of publications in which similar studies were performed on rats (Shogo et al., 2019; Ahn and Kim, 2020; Supruniuk et al., 2020). However, we did not find data on the Shift training influence in old age animals in these publications.

### IL-6 Content in Muscle Tissue in Mice

**FIGURE 5:**
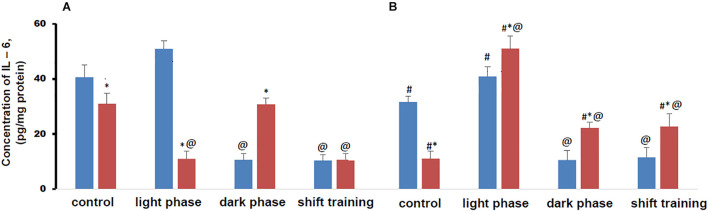
Concentration of interleukin 6 (IL-6) in mice muscle tissue (pg/mg protein). Panel **(A)** shows young-age mice. Panel **(B)** shows old-age mice. Blue columns represent Chow diet and red columns represent High fat diet. Data are presented as the mean ± error of the mean. *Significantly different (*p* < 0.05) from Chow diet. #Significantly different (*p* < 0.05) from young-age mice. @Significantly different (*p* < 0.05) from control.

The concentration of IL-6 in muscle tissue in control groups mice fed a HFD was lower than in the CHD group in both young and old animals (Figure 5).

Regular physical activity in the form of treadmill training led to a significant decrease in the content of IL-6 in muscle tissue in young mice CHD, if the load was applied during the dark phase or the shift training scheme. Exercise during the light phase did not significantly affect the concentration of this myokine in both young and old animals (Figure 5A).

In the HFD group of 20-week-old mice, it was found that the decrease in the IL-6 content in the muscle tissue was facilitated by the loads applied during the light phase and according to the shift training scheme. At the same time, loads during the dark phase did not significantly affect the concentration of this myokine (Figure 5A).

In 48-week-old CHD mice regular exercise in the form of treadmill training had the same effect on the concentration of IL-6 in muscle tissue as in young mice. They led to a significant decrease in the content of IL-6 in muscle tissue if the loads were applied during the dark phase or according to the shift training scheme. Exercise during the light phase did not significantly affect the concentration of this myokine (Figure 5B).

In 48-week-old HFD mice, regular exercise in the form of treadmill training led to an increase in the IL-6 content in muscle tissue in all cases. With the load during the light phase, the gain was twice as high as with the load during the dark phase or with the shift training scheme (Figure 5B).

Metabolic disorders modeled by the HFD significantly altered the IL-6 content in muscle tissue under regular treadmill training. In older mice, these changes were more pronounced. In all groups of animals, the content of the measured myokine largely depended on load during the light phase or load during the dark phase.

Earlier, muscle biopsies from healthy people taken at different time points after running showed that IL-6 mRNA expression was immediately induced by exercise (Louis et al., 2007). Changes in circulating IL-6 levels were noted within 24 h after intense training performed by sedentary volunteers (Garneau et al., 2020). IL-6 protein expression was markedly lower in rat skeletal muscle obese compared to those on a normal diet. Although IL-6 levels were not significantly increased in obese rats after exercise training, the training effect was negligible (Ahn and Kim, 2020). Similarly, previous studies have reported neither significant changes in IL-6 mRNA levels after 11 weeks of training (Haugen et al., 2010), nor in plasma IL-6 levels after 12 weeks of endurance training (Devries et al., 2008).

### IL-15 Content in Muscle Tissue in Mice

**FIGURE 6:**
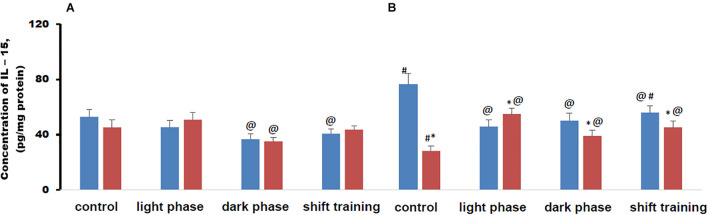
Concentration of interleukin 15 (IL-15) in mice muscle tissue (pg/mg protein). Panel **(A)** shows young-age mice. Panel **(B)** shows old-age mice. Blue columns represent Chow diet and red columns represent High fat diet. Data are presented as the mean ± error of the mean. *Significantly different (*p* < 0.05) from Chow diet. #Significantly different (*p* < 0.05) from young-age mice. @Significantly different (*p* < 0.05) from control.

The concentration of IL-15 in muscle tissue in young HFD mice did not differ from CHD group in all groups. In older mice in the CHD group, the concentration of IL-15 in muscle tissue was higher than in younger mice. In the HFD group, the concentration of this myokine, on the contrary, was significantly lower than in young mice and then in old mice of the CHD group (Figure 6).

Regular physical activity in the form of treadmill training led to a decrease in the content of IL-15 in muscle tissue in young mice CHD, if the load was applied during the dark phase or according to the shift training scheme. At the same time, loads during the light phase did not significantly affect the concentration of this myokine (Figure 6A).

In the HFD group of 20-week-old mice, the concentration of IL-15 in muscle tissue decreased only if the loads were applied during the dark phase. At the same time, loads during the light phase or according to the shift training scheme did not significantly affect the concentration of this myokine (Figure 6A).

In 48-week-old mice, CHD, regular exercise in the form of treadmill training helped to reduce the concentration of IL-15 in muscle tissue, regardless of what time of day they were used. At the same time, in old age HFD mice, regular physical activity in the form of forced running, on the contrary, led to an increase in the content of IL-15 in muscle tissue in all phase groups. If the loads were applied during the light phase, then the concentration of IL-15 was higher than in the CHD group. When applying loads during the dark phase or according to the shift scheme, the training became lower (Figure 6B).

Metabolic disorders modeled by the HFD significantly altered the IL-15 content in muscle tissue under regular treadmill training loads only in 48-week-old mice. The myokine content largely depended on whether the load was applied in the light phase or the dark phase. In the young mice, there were no differences in the concentration of IL-15 in muscle tissue between the HFD and CHD groups.

The studies (Garneau et al., 2020) demonstrated that levels of circulating IL-15 did not increase immediately after a cycling. It was previously found that the concentration of this myokine increased in plasma after an intense cycling exercise (Sabaratnam et al., 2018). In the study, plasma IL-15 levels returned to lower than baseline levels 3 h after recovery. On the other hand, brisk running in healthy subjects caused a gradual increase in muscle IL-15 mRNA expression within 24 h after exercise (Louis et al., 2007). IL-15 can be in the bloodstream in free form or in complex with a soluble form of the alpha subunit of the IL-15 receptor (sIL-15Ralpha), which can change the activity of IL-15 depending on the target cells (Nadeau and Aguer, 2019). It was also found that the lowest levels of IL-15 are found in people who are physically active. Protein levels are higher in sedentary subjects and even higher in sedentary, obese and type 2 diabetics (Perez-Lopez et al., 2018). A study (Ahn and Kim, 2020) demonstrates that an exercise program combining aerobic exercise and resistance exercise activates expression including IL-15 in the skeletal muscle of aging obese rats.

### Leukemia Inhibitory Factor Content in Muscle Tissue in Mice

**FIGURE 7:**
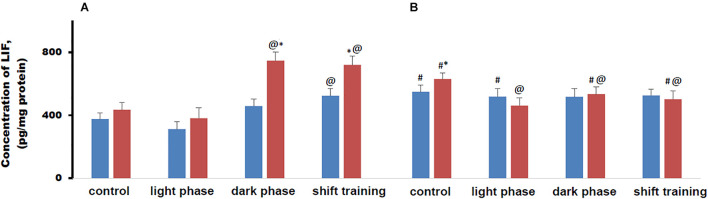
Concentration of leukemia inhibitory factor (LIF) in mice muscle tissue (pg/mg protein). Panel **(A)** shows young-age mice. Panel **(B)** shows old-age mice. Blue columns represent Chow diet and red columns represent High fat diet. Data are presented as the mean ± error of the mean. *Significantly different (*p* < 0.05) from Chow diet. #Significantly different (*p* < 0.05) from young-age mice. @Significantly different (*p* < 0.05) from control.

The concentration of LIF in muscle tissue in young mice fed a HFD did not differ from that in the CHD group. In 48-week-old mice, the concentration of this myokine was higher in both cases, while in the HFD group it was higher than in the CHD group (Figure 7).

Regular physical activity in the form of treadmill training led to a significant increase in the LIF content in muscle tissue in 20-week-old mice when the load was applied during the dark phase or according to the shift training scheme. In the HFD group, the increase in the concentration of this myokine was significantly greater than in the CHD group. The loads during the light phase did not significantly affect the concentration of this myokine (Figure 7A).

In 48-week-old mice CHD mice, LIF concentration in muscle tissue did not change regardless of the treadmill training regimen. In old age HFD mice, regular exercise in the form of treadmill training led to a decrease in the LIF content in muscle tissue in all cases. If the loads were applied during the dark phase or according to the shift training scheme, the concentration of LIF in muscle tissue was lower than in 20-week-old mice (Figure 7B).

Metabolic disorders modeled by the HFD significantly modified the production of this myokine during regular exercise in the form of forced running. The changes depended to a large extent on the age of the animals and on the scheme of applying the loads. In old animals, the response to physical activity from the LIF was recorded only in the HFD group.

Leukemia inhibitory factor is a myokine that belongs to the IL-6 superfamily. It has several biological functions, stimulating the formation of platelets and neurons, the proliferation of hematopoietic stem cells, and the formation of bone tissue (Metcalf, 2003). It should be noted that LIF has a very short serum half-life (6–8 min) (Hilton et al., 1991). It makes it difficult to detect. For this reason, the patterns of LIF expression and secretion during exercise are not fully understood (Huh, 2018; Gomarasca et al., 2020).

It should be noted that an increase in the number of transcripts of various secreted factors, including IL-15 and LIF, does not always lead to a proportional increase in systemic concentrations (Catoire et al., 2014; Landers-Ramos et al., 2014). Typically, systemic cytokine responses are more pronounced after exercise, which causes more muscle damage (Paulsen et al., 2012).

### CXCL1 Content in Muscle Tissue in Mice

**FIGURE 8:**
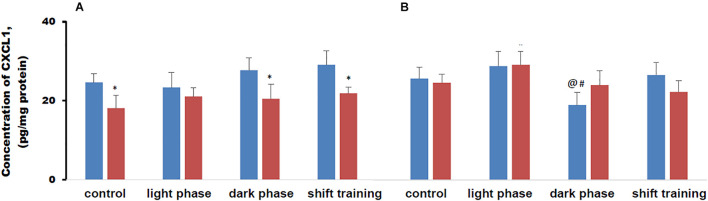
Concentration of chemokine ligand 1 (CXCL1) in mice muscle tissue (pg/mg protein). Panel **(A)** shows young-age mice. Panel **(B)** shows old-age mice. Blue columns represent Chow diet and red columns represent high fat diet. Data are presented as the mean ± error of the mean. *Significantly different (*p* < 0.05) from Chow diet. #Significantly different (*p* < 0.05) from young-age mice. @Significantly different (*p* < 0.05) from control.

The concentration of CXCL1 in muscle tissue in mice fed a HFD was lower than in the CHD group in 20-week-old mice. In 48-week-old mice, there were no differences between the groups. There were no differences between the values of the CXCL1 concentration in muscle tissue in young and old mice (Figure 8).

Regular physical activity in the form of treadmill training did not affect the content of CXCL1 in muscle tissue in 20-week-old mice in the CHD and HFD groups, for all load regimens. When using loads during the dark phase or shift training, the concentration of CXCL1 in muscle tissue in the HFD group was lower than in the CHD group. When using the load during the light phase, there was no difference between the groups (Figure 8A).

In 48-week-old mice, CHD, regular exercise in the form of treadmill training did not affect the concentration of CXCL1 in muscle tissue in almost all cases. The exception was a group of old mice exposed to stress during the dark phase. In this group, treadmill training resulted in a CXCL1 concentration decrease in muscle tissue in comparison with animals that were not exposed to stress and compared with young mice exposed to the same pattern (Figure 8B).

Metabolic disturbances modeled by a HFD contributed to a decrease in CXCL1 content in muscle tissue in young animals. Practically in all groups of animals, the content of the indicated myokine did not change with the use of forced running.

It has been shown that some of the chemokines are produced by muscle cells during contraction (Pedersen et al., 2011). Muscle electrical stimulation *in vitro* promotes the accumulation of chemokines in muscle tissue, while the extracellular Ca2 + chelator EGTA reduced this effect by half (Nedachi et al., 2009). The production of CXCL1 by muscles and its content in blood serum significantly increases in response to a single physical exercise (Pedersen et al., 2012). It can be assumed that these effects are manifested only during acute single physical activity and are not recorded during long-term chronic exposure.

It has been suggested that catecholamines promote the release of IL-6 and CXCL1 in muscle cells during EPS (Mattingly et al., 2017). A relationship was found between the amounts of IL-6 and CXCL1 released in individual muscles in EPS. These results suggest that these cytokines are either somehow regulated by similar transcriptional, translational, or secretory pathways, or the secretion of one cytokine affects the secretion of another (Mattingly et al., 2017).

Our results are partially consistent with the data (Ahn and Kim, 2020), which showed that the level of chemokines was significantly lower in skeletal muscle in rats fed a high-fat diet compared to the group with a normal diet. However, after completing a 12-week exercise program, the CXC was significantly higher in the HFD shift training group (running and resistance exercise) according to Ahn and Kim (2020) compared to the HFD group without exercise.

It is important to note that in our study, the animals were sacrificed 24 h after the last exercise. In the literature, there is a different nature of myokine secretion, depending on the time elapsed after exercise. The differences between the results of studies in mice, rats and in humans are also described. Thus, in an experiment on mice, an increase in the level of CXCL1 in muscles (m. Tibialis cranialis, m. Gastrocnemius, m. Soleus) was shown immediately after exercise (1 h of swimming) by 50%. After 2 h, the protein level returned to the initial level, and after 5 h it decreased by 50% relative to the basal concentration (Pedersen et al., 2011). In men, the LIF mRNA level increased 4.5 times 1.5 h after pedaling on a bicycle ergometer in the vastus lateralis muscle and remained for 3 h (Broholm et al., 2008). In studies on satellite cells from the vastus lateralis muscle after electrical stimulation after 24 h, an increase in the level of LIF mRNA was noted in comparison with the basal one (Broholm et al., 2011). Exercise caused a decrease in LIF mRNA expression in wild-type muscle in mice. Similar suppression of LIF mRNA after exercise was observed in the tibialis anterior muscle and diaphragm of MDX mice at three and 6 weeks of age, respectively, compared with wild-type controls (Hunt et al., 2011).

## Discussion

The features of physical activity effect on the myokines content in muscle tissue in mice may be associated with differences in the cellular composition of the muscles. Skeletal muscle is heterogeneous. In addition to myocytes, it contains a number of cells: fibroblasts, pericytes, and adipocytes. The contribution of these cells to the total volume of cytokine production has not been sufficiently determined (Peake et al., 2015). The relative abundance of these cells, as well as their involvement in exercise-induced cytokine production, may differ in healthy animals and in animals with a metabolic disorder model. In addition to tissue heterogeneity, it has been shown that skeletal myocytes can be subdivided into three different phenotypes, each with its own characteristics of bioenergetic mechanisms. In DM II “white” (oxidative) muscle fibers predominate and the proportion of “red” (glycolytic) fibers decreases (Fitts and Widrick, 1996).

Another mechanism providing the described differences may be associated with the features of transcriptional mechanisms. It has been shown that transcriptional changes during muscle contraction are most pronounced in rapidly contracting type IIa muscle fibers (Raue et al., 2012). The proportion of these fibers in patients with DM II increases, which is reflected in the intensity of myokine production.

Of particular interest is the connection between the metabolic and secretory effects of physical exercises and the period of their performance during the day (Savikj et al., 2019). A growing body of evidence suggests that cellular metabolism exhibits consistent diurnal fluctuations (Domin et al., 2021). Indeed, several metabolic pathways are directly controlled by the circadian clock through genetic and epigenetic regulation of metabolic enzymes (Bass and Takahashi, 2010; Masri and Sassone-Corsi, 2014; Berger and Sassone-Corsi, 2016). Thus, the daily timing of food intake, physical activity, sleep and drug treatment can provide a variety of metabolic and physiological responses and can affect the pathogenesis of the disease. This concept has generated interest in research aimed at determining the optimal timing of physical activity, as well as therapeutic treatment regimens to improve glucose and energy homeostasis (Belancio et al., 2015).

The study (Shogo et al., 2019) shows that exercise time can enhance the metabolic effect of exercise. At the same time, exercise during the early resting phase stimulates daily fluctuations in genes and metabolites associated with carbohydrate metabolism, but weaken the daily rhythm of genes and metabolites associated with glycerol metabolism. The rhythmic fluctuations in genes and metabolites associated with carbohydrate metabolism are lost after exercise during the early active phase. In addition to altered cyclic regulation of metabolic pathways in skeletal muscle, systemic oscillatory energy homeostasis exhibits various adaptations unique to daily exercise times. These data suggest that exercise modulates the molecular clock (Schroder et al., 2013). However, their effect on the metabolic cycle seems to depend on the time of day.

Also (Shogo et al., 2019) found a time-dependent effect of exercise on the activation of the transcription factor HIF1a, which is responsible for hypoxia. The role of HIF1a in the regulation of the circadian clock is known (Adamovich et al., 2017; Peek et al., 2017; Wu et al., 2017). HIF1a may be a key link in mechanisms that induce time-specific modifications to the temporary control of metabolic pathways by exercise (Shogo et al., 2019).

The HIF1a targets in combination with CBP-p300 and associated with histone hyperacetylation are highly enriched genes regulated exclusively by exercise in the early active vs. early rest phase. Since HIF1a has been associated with a molecular clock (Adamovich et al., 2017; Peek et al., 2017; Wu et al., 2017), circadian variations in HIF1a activity can lead to a distinct effect of exercise in the early active phase on its descending signals. Skeletal muscle metabolic analysis (Shogo et al., 2019) shows carbohydrate depletion only after the start of active exercise. In addition to carbohydrates, skeletal muscle uses a variety of energy sources during endurance exercise, including lipids, amino acids, and ketone bodies (Overmyer et al., 2015; Evans et al., 2017; Lundsgaard et al., 2018). Exercise in the early active phase significantly increases the level of muscle acyl carnitines simultaneously with genes involved in fatty acid oxidation and ketolysis (Mika et al., 2019; Shogo et al., 2019).

The work (Ezagouri et al., 2019) identified metabolic pathways that are differently activated during exercise, depending on the time of day. Notably, ZMP (5-aminoimidazole-4-carboxamide ribonucleotide), an endogenous AMPK activator, is exercise-induced depending on the time of day and is able to regulate key steps in glycolysis and fatty acid oxidation. Thereby, potentially increase the working capacity. It can be assumed that exercise time is an important modifier of exercise tolerance and associated metabolic pathways. Modification of these mechanisms under the influence of metabolic disorders and physical exertion is of considerable interest, since they are a promising way of influencing metabolic processes at the cellular and systemic levels. This is very important for the search for new ways to correct metabolic disorders.

## Conclusion

Treadmill training influences the content of cytokines in the skeletal muscles of mice with metabolic disorders caused by a 16-week high-fat diet. This effect depends on both the animal’s age and the time of day in which the run is performed. Thus, in 20-week-old animals, the effect of exercise did not depend on the training regimen; at the same time, in 48-week-old animals, the decrease in body weight in mice with a shift training regimen was more pronounced.

High fat diet affects muscle myokine levels. The content of all myokines, except for LIF, decreased, and the concentration of CLCX1 decreased only in young animals in response to HFD. Training on a treadmill caused multidirectional changes in the concentration of myokines in muscle tissue. The IL-6 content was changed the most. These changes were observed in all groups of animals. To the greatest extent, the changes depended on the training time. The effect of physical activity on the content of IL-15 in skeletal muscle tissue was observed mainly in 48-week-old mice. In 20-week-old animals, physical activity influence to increase in muscle LIF concentration during the dark or shift training regimen. In the HFD group, this effect was significantly more pronounced. The content of CXCL1 did not change when using a treadmill in almost all groups of animals.

It can be assumed that physical activity during different time a day is a promising way to influence metabolic processes at the cellular and systemic levels. It can be important for the searching a new ways to correct metabolic disorders.

### Limitations

The main limitation of this study results is associated with the differentiation of sources of cytokine production in muscle tissue. These sources can be both muscle cells and other types of cells, including macrophages infiltrating muscle tissue or other immune cells. However, numerous studies, including our own studies on the culture of myoblasts, the links to which are given above, directly confirm a significant increase in the production of cytokines by muscle cells during exercise. It is obvious that the contribution of the “muscle component” to the production of cytokines in muscle tissue is significant.

## Data Availability Statement

The original contributions presented in the study are included in the article/supplementary material, further inquiries can be directed to the corresponding author/s.

## Ethics Statement

The animal study was reviewed and approved by Bioethics Commission of the Biological Institute of Tomsk State University (Protocol No. 32 dated 02.12.2019).

## Author Contributions

AZ, ED, AC, and LK contributed to the development of the research concept and methodology, analysis of the results, and article writing and editing. TK, KM, AO, AK, and GK were directly involved in the implementation of experimental studies. All authors contributed to the article and approved the submitted version.

## Conflict of Interest

The authors declare that the research was conducted in the absence of any commercial or financial relationships that could be construed as a potential conflict of interest.

## Publisher’s Note

All claims expressed in this article are solely those of the authors and do not necessarily represent those of their affiliated organizations, or those of the publisher, the editors and the reviewers. Any product that may be evaluated in this article, or claim that may be made by its manufacturer, is not guaranteed or endorsed by the publisher.

## Footnotes

1
https://www.biopro.ru/catalog/dlya_gryzunov/486/
